# Preparation and Hydrogelling Performances of a New Drilling Fluid Filtrate Reducer from Plant Press Slag

**DOI:** 10.3390/gels8040201

**Published:** 2022-03-23

**Authors:** Wenjun Long, Xialei Zhu, Fengshan Zhou, Zhen Yan, Amutenya Evelina, Jinliang Liu, Zhongjin Wei, Liang Ma

**Affiliations:** 1 Beijing Key Laboratory of Materials Utilization of Nonmetallic Minerals and Solid Wastes, National Laboratory of Mineral Materials, School of Materials Science and Technology, China University of Geosciences (Beijing), No. 29 Xueyuan Road, Haidian District, Beijing 100083, China; longwj@cugb.edu.cn (W.L.); zhuxialei1@163.com (X.Z.); yanzh@bjeea.cn (Z.Y.); evelinametine288@gmail.com (A.E.); 2003190045@cugb.edu.cn (J.L.); weizhongjin@hotmail.com (Z.W.); maxliang@cugb.edu.cn (L.M.); 2 Goldwind Environmental Protection Co., Ltd., No. 26 Kechuang 13th Street, Daxing District, Beijing 100176, China; 3 Beijing Education Examination Authority, No. 9 Zhixin East Road, Haidian District, Beijing 100083, China

**Keywords:** plant press slag (PPS), carboxymethylation, mixture of carboxymethyl cellulose and carboxymethyl starch (CMCS), filtrate reducer, drilling fluids

## Abstract

Plant press slag (PPS) containing abundant cellulose and starch is a byproduct in the deep processing of fruits, cereals, and tuberous crops products. PPS can be modified by using caustic soda and chloroacetic acid to obtain an inexpensive and environmentally friendly filtrate reducer of drilling fluids. The optimum mass ratio of m_NaOH_:m_MCA_:m_PPS_ is 1:1:2, the optimum etherification temperature is 75 °C, and the obtained product is a natural mixture of carboxymethyl cellulose and carboxymethyl starch (CMCS). PPS and CMCS are characterized by using X-ray diffraction, scanning electron microscopy, Fourier transform infrared spectroscopy, thermogravimetric, X-ray photoelectron spectroscopy, and elemental analysis. The filtration loss performance of CMCS is stable before and after hot-rolling aging at 120 °C in 4.00% NaCl and saturated NaCl brine base slurry. The minimum filtration loss value of CMCS is 5.28 mL/30 min at the dosage of 1.50%. Compared with the commercial filtrate reducers with a single component, i.e., carboxymethyl starch (CMS) and low viscosity sodium carboxymethyl cellulose (LV-CMC), CMCS have a better tolerance to high temperature of 120 °C and high concentration of NaCl. The filtration loss performance of low-cost CMCS can reach the standards of LV-CMC and CMS of the specification of water-based drilling fluid materials in petroleum industry.

## 1. Introduction

The comprehensive utilization of agricultural waste materials has gained much popularity in recent years. Agricultural waste materials show numerous advantages such as cost effectiveness and reducing environmental hazards [1,2,3,4]. Many agricultural waste materials are produced because of the rapid development of agriculture in China. For example, the corn production was about 2.16 × 10^8^ tons in 2017 in China, and about 3.5 × 10^7^–3.9 × 10^7^ tons corncob could be obtained from the corn production [5]. Agricultural waste material contains starch, cellulose, hemi-cellulose, etc. It could ferment and deteriorate easily and would cause environmental pollution. Some of them have been separated and purified to product feed additive [6], prebiotic food [7], protein feed [8], etc. Additionally, some of them have even been used to produce bioethanol [9]. However, its popularization and application are difficult to realize due to the complex process, huge investment, and low economic effectiveness.

Many studies have addressed the subject of using cellulosic agricultural wastes in oil field applications and a wide variety of cellulosic agricultural waste materials are available for using as drilling fluids additives, including corncob, rice husk, bamboo, and so on [10]. Some of them showed a good performance such as Tapioca starch [11] and data seed powder (DSP) [12]. However, there are still many engineering problems in the direct use of agricultural wastes, such as too much added dosages needed in the drilling fluids [13], some samples might cause a negative impact to the rheological properties of the drilling fluids [14,15]. In a nutshell, the added value of using agricultural wastes directly as drilling fluid additives is low. However, through appropriate low-cost chemical modification techniques, the performances and added value of additives derived based on the agricultural wastes can be greatly improved [16,17]. This is very beneficial for both additive producers and oilfield users.

There is no well drilling without the use of drilling fluids. Drilling fluids have many functions, including carrying and suspending cuttings, balancing stratum pressure, and reducing the filtration loss value, etc. Water-based drilling fluid is a widely used drilling fluid and it is a kind of suspension composed of bentonite, water, chemical additives, etc. Filtration control is an important property of a drilling fluid, particularly when drilling through permeable formations, where the hydrostatic pressure exceeds the formation pressure. It is important for a drilling fluid to quickly form a filter cake to effectively minimize fluid loss. Fluid loss control additives, also called filtrate-reducing agents or filtrate reducer, is crucial to the performance control of drilling fluid, which is mainly reflected in four aspects: firstly, enhancing the borehole stability for maintain normal and safe drilling in water sensitive formation and crushed formation; secondly, protecting the formation damage caused by filtrate loss immersion; thirdly, maintaining the colloid stability of drilling fluid system to suspend and carry drilling cutting particles; and lastly, aiding the bit tool cut rock while water jet drilling to improve the rate of penetration (ROP). The consumption of filtrate reducers accounts for about 10% of the whole drilling fluid additives in amount, but its cost accounts for about 30%, so filtrate reducer is the most important drilling fluid additive. Commonly used filtrate reducers include modified starch, modified cellulose, humic acid derivatives, low molecular polyacrylamide-like copolymers, partially hydrolyzed polyacrylonitrile, synthetic water-soluble resin, and so on [17,18,19,20].

Conventional chemical additives used in drilling fluids have been found to have a negative impact on the environment and human health, and the most commercially available chemical additives are not biodegradable [21,22]. Recently, nanomaterials have been used as drilling fluid additives, improving their rheology, lubricity, and filtration property [23,24,25]. However, due to their poor dispersion ability, low performance stability, and relatively high prices, their usage is limited [26,27]. Therefore, there is a great need for new widely used biodegradable environmentally friendly drilling fluid additives, which can help control the performances of drilling fluids, causing little impact on the environment and to the health of workers [15]. CMC (carboxymethyl cellulose) and CMS (carboxymethyl starch) are typical biodegradable water-soluble polymers. CMC is often called “the monosodium glutamate of industry”. CMC and CMS can be used in oil and gas well drilling [28,29], drug release [30,31,32,33,34], food industry [35,36], etc. When used in well drilling, CMC is roughly divided into two classes, i.e., HV-CMC (high-viscosity sodium carboxymethyl cellulose) and LV-CMC (low-viscosity sodium carboxymethyl cellulose). HV-CMC is mainly used as a natural tackifier [37], while LV-CMC is the most widely used filtrate reducer in water-based drilling fluids [38,39]. However, due to the rising price of short linters for CMC production, preparing CMC using non-cotton cellulose has become increasingly popular. Main alternative raw materials include waste disposable paper cup [40], corncob residue [41], cassava residue [42], etc. CMS is close to LV-CMC in almost all performances except for salt resistance and is often used as drilling fluid loss agent to replace LV-CMC. The reaction mechanism of using cellulose and starch to prepare filtrate reducers is the same, but the reaction conditions are different [43,44].

Plant press slag (PPS) is a byproduct in the deep processing of natural products and it contains starch, cellulose, pectin, etc. It was treated by dehydration, drying, and then processed into powdery or granular fodder. The economic effectiveness of dealing with PPS in this way was low. Because of a high starch and cellulose content in PPS, we can synthesize a mixed product of LV-CMC and CMS by alkalizing and etherifying the PPS, which was a natural mixture of carboxymethyl cellulose and carboxymethyl starch (CMCS). We determined the optimal reaction conditions of CMCS, evaluated its applied performances, and characterized its structure. We compared CMCS with CMS and LV-CMC in terms of the filtration loss performance, tolerance to high temperature and salt. We also compared the filtration loss performance of CMCS with the standards of LV-CMC and CMCS of the specification of drilling fluid materials.

## 2. Results and Discussion

### 2.1. Structural Characterizations

We used a X-ray diffraction analyzer, scanning electron microscope, infrared spectrometer, and thermogravimeter to test, analyze, and compare PPS and CMCS. CMCS samples were prepared according to the optimal mass ratio of m_NaOH_:m_MCA_:m_PPS_ at 1:1:2 and etherification reaction at 75 °C for 1 h.

#### 2.1.1. Elemental Analysis (EA)

The contents of carbon, hydrogen, and oxygen of PPS and CMCS were determined by using an organic element analyzer. According to the molecular formula of cellulose and starch, the theoretical content ratio of C/H is 7.2, and the ratio of C/O is 0.9. Due to the presence of impurities in PPS, the ratio of C/H and C/O were relatively low than the theoretical ratio. The ratio of C/H and C/O of chloroacetic acid is 8.0 and 0.75, respectively. Therefore, the ratio of C/H should increase, while the ratio of C/O should decrease theoretically after modification. As shown in Table 1, the experimental results were in good agreement with theory, indicating the success of the modification reaction of PPS to a certain extent.

#### 2.1.2. XRD Characterization

**Figure 1:**
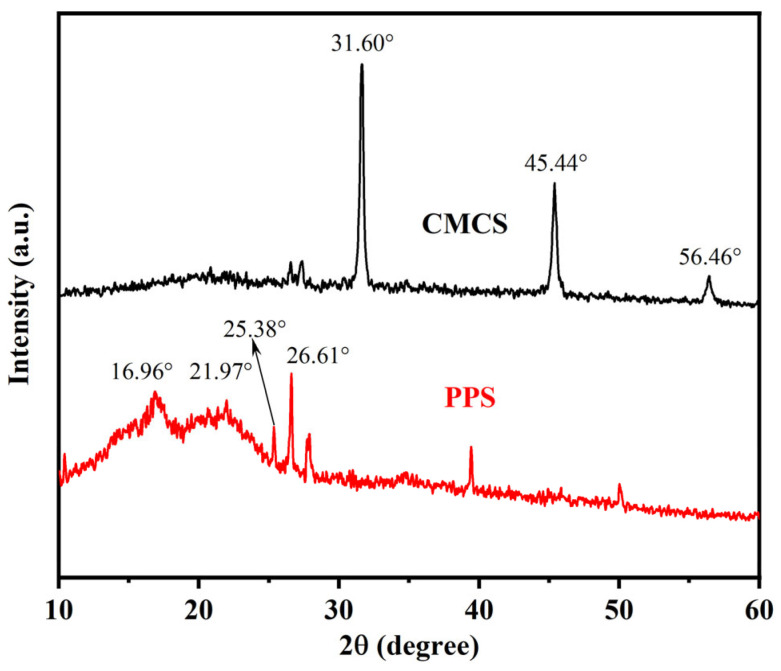
X-ray diffraction spectrograms of PPS and CMCS.

An X-ray diffractometer was used to analyze PPS and CMCS, respectively (Figure 1). Starch has three crystal structures of A, B, and C, generating three types of XRD patterns [29]. From the diffractogram of PPS, PPS has C-type diffraction pattern, which is a mixture of A-type and B-type. Obvious diffraction peaks at around 2θ = 16.96°, 21.97°, 25.38°, and 26.61° could be observed for PPS, indicating its semi-crystalline structure [35,36]. The peaks were broad due to the crystallites of the cellulose in PPS [40]. After the alkalization and etherification in the process of carboxymethylation, the peaks at 2θ = 16.96°, 21.97°, 25.38°, and 26.61° in the diffractogram of CMCS disappeared completely, revealing the crystalline structures of cellulose and starch in PPS were destroyed and the crystallinity decreased dramatically [45,46]. Furthermore, new peaks were observed at 2θ = 31.60°, 45.44°, and 56.46°, which were the characteristic diffraction peaks of NaCl crystal. This was attributed to the crystallinity of the remaining NaCl in CMCS [40,47]. The change in the XRD pattern of the sample after the modification was due to the destruction of the hydrogen bond structure of the cellulose and starch in the PPS under the action of strong alkaline, heat, and mechanical forces, resulting in a decrease in its crystallinity, which is conducive to improving the hydrophilicity of the sample and its filtrate reduction performance.

#### 2.1.3. SEM Characterization

**Figure 2:**
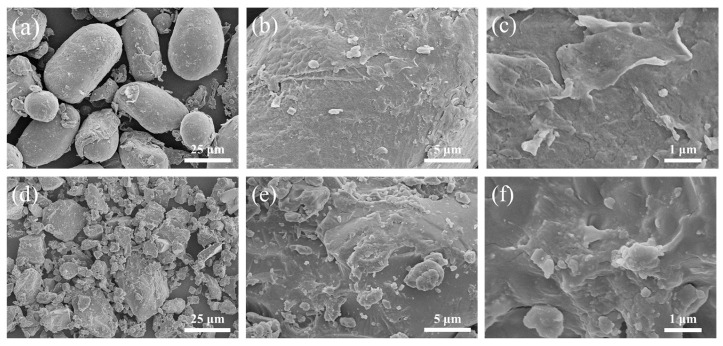
Scanning electron microscope images of PPS and CMCS. (**a**) PPS × 1000; (**b**) PPS × 5000; (**c**) PPS × 20,000; (**d**) CMCS × 1000; (**e**) CMCS × 5000; (**f**) CMCS × 20,000. CMCS was obtained by carboxymethylation when the mass ratio of m_NaOH_:m_MCA_:m_PPS_ was 1:1:2 and etherification temperature was 75 °C.

As shown in Figure 2, the morphology of PPS particles changed significantly after the modification. The original morphology of PPS particles was in an irregular elliptical egg shape, with a smooth and flat surface, and the diameter of those “eggs” were estimated in the range of approximately 10–30 μm (Figure 2a–c). However, the surface of CMCS became rough, with wrinkles, of which holes and cracks were obviously presented (Figure 2e,f). The hydroxyl group in PPS reacted with sodium hydroxide, part of the hydrogen bonds of PPS were broken, resulting in the decrease in its crystallinity and the formation of holes and cracks under the action of strong alkali, heat, and mechanical forces. Moreover, the diameter of the PPS particles was reduced (Figure 2d). Due to the smaller particles, holes and cracks on its surface, the contact area between PPS and etherification agent was increased, which is beneficial to the etherification reaction [36,43].

#### 2.1.4. Infrared Spectroscopy Characterization

**Figure 3:**
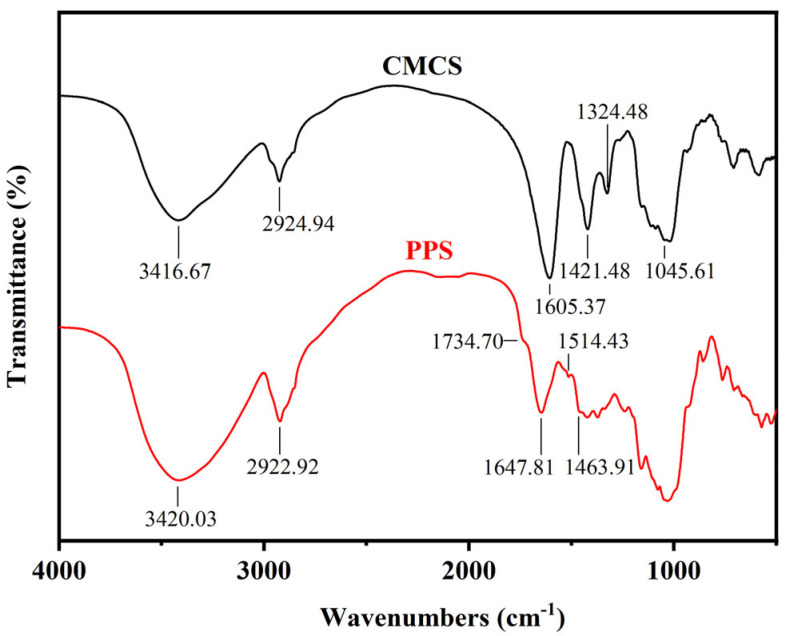
FT-IR spectrograms of PPS and CMCS.

FTIR spectra was used to analyze the chemical groups of PPS and CMCS (Figure 3). The peaks at 3420.03 cm^−1^ and 2922.92 cm^−1^ in FTIR spectra of PPS were due to the stretching vibration of hydroxyl groups and methylene groups [41]. The peak at 1734.70 cm^−1^ originating from the carbonyl stretching vibrations of hemi-cellulose in PPS [48]. The peaks at 1647.81 cm^−1^, 1514.43 cm^−1^, and 1463.91 cm^−1^ were presented in FTIR spectra of PPS, which were due to the aromatic vibrations of the aromatic skeleton of lignin [40,49]. Compared with PPS, the peak at 3416.67 cm^−1^ was also due to the stretching vibration of hydroxyl groups, but the intensity of the signal was slightly reduced, indicating that part of the hydroxyl groups reacted during the modification. The new vibration peaks at 1605.37 cm^−1^ in FTIR spectra of CMCS were attributed to the asymmetrical stretching vibration of C=O in –COO groups. The new peaks at 1421.48 cm^−1^ were the characteristic absorption peak of the long-chain crystalline carboxylate [17,40,49]. Moreover, the peaks at 1324.40 cm^−1^ and 1045.61 cm^−1^ were attributed to the bending vibrations of –OH group and the stretching vibration of CH–O–CH_2_ [43]. These results showed that carboxymethyl groups were successfully grafted on the molecular chain of cellulose and starch in PPS by etherification [41,50].

#### 2.1.5. Thermogravimetric Characterization

**Figure 4:**
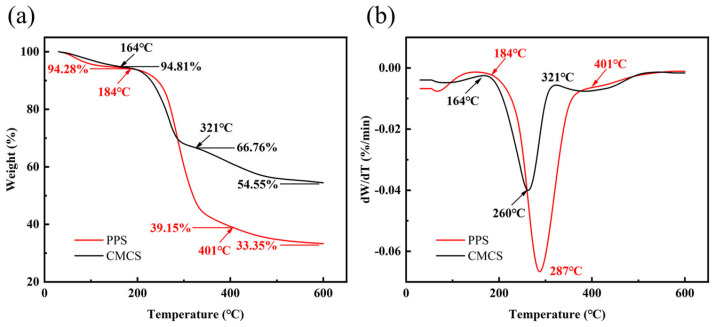
The thermal properties of PPS and CMCS. (**a**) Thermogravimetric (TG) analysis curves of PPS and CMCS; (**b**) Differential thermogravimetric (DTG) curves of PPS and CMCS.

The weight loss of the samples as a function of temperature (TG curves) is shown in Figure 4a, the derivate weight loss curves (DTG curves) were obtained by calculating the first derivative of TG (Figure 4b). Thermal behavior of PPS and CMCS were examined by the study of TG, DTG thermograms. The lost weight of the samples before 150 °C was attributed to the evaporation of its remaining free water and bound water on its surface [51]. The initial decomposition temperature of PPS was about 184 °C and the weight of PPS at 184 °C was 94.28%. The thermogravimetric curve of PPS gradually became flat at about 401 °C with the weight of 39.15%, losing 55.13% of its weight in this thermal decomposition process and the weight of 33.35% of PPS remained when the temperature reached at 600 °C. However, the initial decomposition temperature of CMCS obtained by carboxymethylation was about 164 °C, which was about 20 °C lower than that of PPS and the weight of CMCS was 94.81% at 164 °C. This is because the crystal structure of cellulose and starch in PPS was destroyed during the preparation of CMCS, the CMCS particles were smaller, and the internal space of CMCS became loose, which led to a poor thermal stability of CMCS [52]. The thermogravimetric curve of CMCS became gradually slower at about 321 °C with the weight of 66.67%, and 28.05% of its weight lost in this decomposition process. The weight of 54.55% of CMCS remained after thermal decomposition (Figure 4a). The maximum thermogravimetric rate of PPS and CMCS was at about 287 °C and 260 °C, respectively. The thermogravimetric interval span of PPS was wider than that of CMCS, and the decomposition of CMCS was relatively slow resulting from the etherification of PPS (Figure 4b).

The TG curve indicated the residual mass of CMCS (54.55%) after thermal decomposition was much more than that of PPS (33.35%). There were two reasons for this result. One of the reasons is because after etherification, carboxymethyl groups with high temperature resistance were successfully introduced into the molecular chains of PPS, which significantly improved the thermal stability of CMCS [53,54]. Moreover, the sodium ions introduced during the modification process also attributed to the higher residual mass of CMCS compared with PPS after the thermal decomposition [40].

#### 2.1.6. XPS Analysis

**Figure 5:**
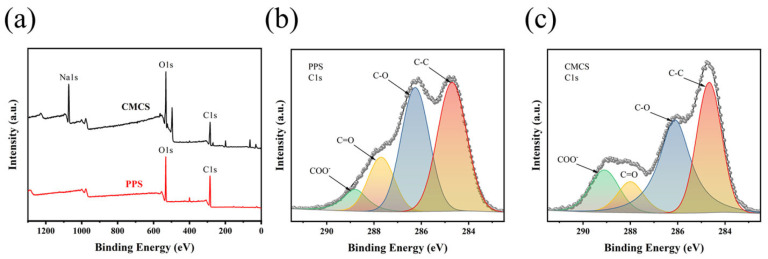
XPS spectra of PPS and CMCS. (**a**) Comparisons of low-resolution mode spectra of PPS and CMCS; (**b**) High-resolution mode XPS spectra of the C1s peaks in PPS; (**c**) High-resolution mode XPS spectra of the C1s peaks in CMCS.

The XPS spectra of PPS before and after modification were investigated to trace and compare the structural differences, the corresponding results are presented in Figure 5. As is shown in Figure 5a. the low-resolution mode spectra of PPS and CMCS indicate that PPS has two sharp peaks (O1s, C1s), whereas CMCS has three peaks (Na1s, O1s, C1s).

The changes in carbon relative content in different combination states were revealed by the high-resolution C1s peak of PPS and CMCS (Figure 5b,c). The C1s peak was fitted into four distinct peaks that corresponded to C_1_ (C–C, 284.7 eV), C_2_ (C–O, 286.3 eV), C_3_ (C=O, 287.7 eV), and C_4_ (O=C–O, 288.8 eV). The presence of C_3_, C_4_ in PPS was related to its pectin and lignin components. The relative content of C_2_ of PPS was decreased, while the composition of C_4_ was increased after modification. The above results suggested that the carboxyl functional groups were successfully introduced into the molecular chain of PPS after alkalization and etherification reaction, which is consistent with previous studies [55,56,57,58].

### 2.2. Optimization of Experimental Parameters for Carboxymethylation

The optimized experimental parameters can be obtained according to the minimum value of the filtration loss of the saturated NaCl water-based drilling fluid with the sample dosage of 1.00% (4.00 g sample was added in 400.00 mL brine base slurry) after curing at room temperature for 16 h [59].

#### 2.2.1. Effect of NaOH Dosage on Filtration Loss Performance of CMCS

**Figure 6:**
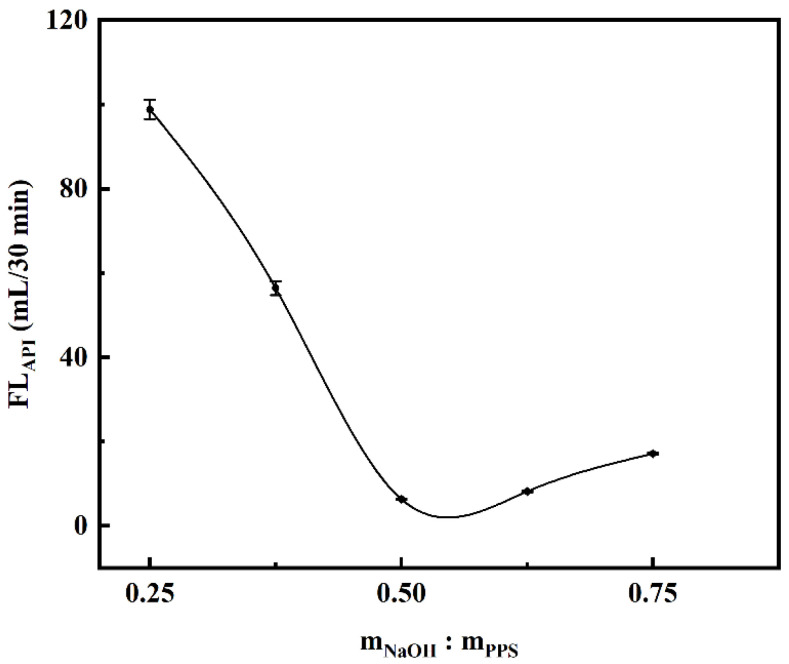
Effect of NaOH on saturated NaCl brine base slurry filtration loss performance of CMCS. (The mass ratio of m_MCA_:m_PPS_ was 0.50 and the etherification temperature was 75 °C).

The alkalized PPS samples were obtained by adding sodium hydroxide solution to the ethanol aqueous solution of PPS, wherein the mass ratio of m_NaOH_:m_PPS_ was 0.250, 0.375, 0.500, 0.625, and 0.750, respectively. The controlled experimental variables for this parameter were the mass ratio of m_MCA_:m_PPS_ which was 0.50 and the etherification temperature which was 75 °C. At the sample dosage of 1.00%, the API filtration loss tests of these samples in saturated NaCl brine base slurry were carried out after curing at room temperature for 16 h. As the amount of NaOH increased, the API filtration loss decreased at first, and later reached the minimum value of 6.32 mL when m_NaOH_:m_PPS_ was 0.50 (Figure 6).

When the mass ratio of m_NaOH_:m_PPS_ was lower than 0.50, the degree of swelling of PPS was insufficient, which was not conducive to the subsequent etherification reaction of MCA and PPS, resulting in lower etherification degree and poor filtration loss performance. When the mass ratio of m_NaOH_:m_PPS_ was higher than 0.50, the PPS could be degraded by excessive NaOH, and excessive NaOH would react with the etherifying agent (monochloroacetic acid), producing byproducts such as sodium glycolate and lowering the efficient of etherifying agent, which caused a decreased etherification degree and inferior filtration loss performance of the sample [16,43].

#### 2.2.2. Effect of Etherifying Agent Dosage on Filtration Loss Performance of CMCS

**Figure 7:**
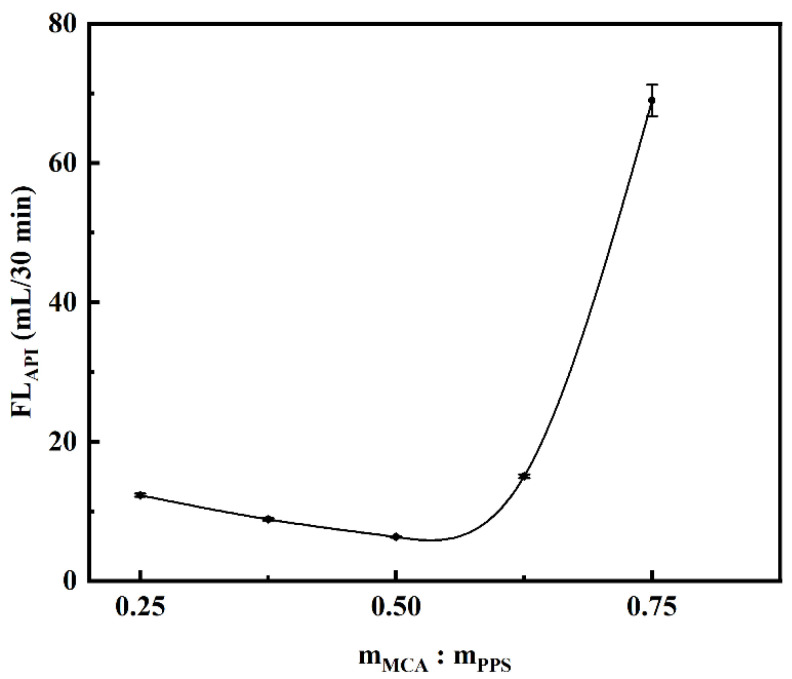
Effect of MCA on saturated NaCl brine base slurry filtration loss performance of CMCS. (The mass ratio of m_NaOH_:m_PPS_ was 0.50 and the etherification temperature was 75 °C).

The etherified samples were prepared by adding monochloroacetic acid (MCA) to the ethanol aqueous solution of alkalized PPS, wherein the mass ratio of m_MCA_:m_PPS_ was 0.250, 0.375, 0.500, 0.625, and 0.750, respectively. For this experimental parameter, the controlled conditions were the mass ratio of m_NaOH_:m_PPS_ which was 0.50 and the etherification temperature which was 75 °C. After curing at room temperature for 16 h, the API filtration loss tests of these samples in saturated NaCl brine base slurry were executed and the sample dosage was 1.00%. As the amount of MCA increased, the API filtration loss decreased at first and then increased, and it reached the minimum value of 6.32 mL when m_MCA_:m_PPS_ was 0.50 (Figure 7). When the mass ratio of m_MCA_:m_PPS_ was lower than 0.50, the inadequate etherification degree resulted in a reduced amount of carboxyl functional groups on CMCS, which led to a poor salt tolerance property and filtrate reduction effect of the sample. The increase in the MCA concentration made it easier to enter the interior of PPS for reaction. However, when the mass ratio of m_MCA_:m_PPS_ was higher than 0.50, the excessive MCA could affect the PH value of the solution and easily aggravate side reactions, which would reduce the etherification efficiency and filtrate reduction effect [60].

#### 2.2.3. Effect of Etherification Temperature on Filtration Loss Performance of CMCS

**Figure 8:**
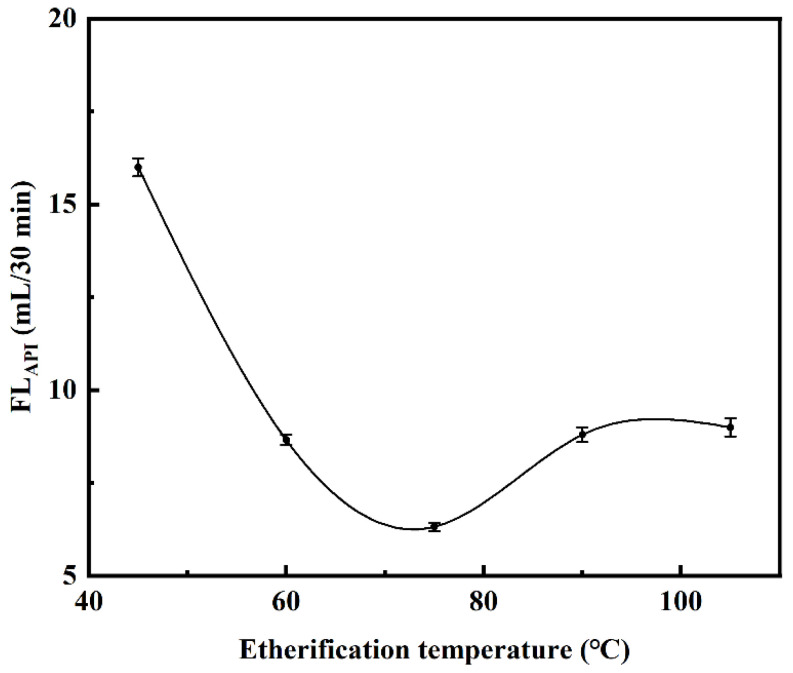
Effect of etherification temperature on saturated NaCl brine base slurry filtration loss performance of CMCS. (The mass ratio of m_NaOH_:m_MCA_:m_PPS_ was 1:1:2).

The etherification was carried out at different temperatures, including 45 °C, 60 °C, 75 °C, 90 °C, and 105 °C. The mass ratio of m_NaOH_:m_MCA_:m_PPS_ was kept constant at 1:1:2 for this experimental parameter. At the sample dosage of 1.00%, the API filtration loss tests of these samples in saturated NaCl brine base slurry were implemented after curing at room temperature for 16 h. As the etherification temperature increased, the filtration loss value of sample decreased first and then increased, cause the increasing in temperature between 45 °C and 75 °C improved the swelling of PPS particles and the etherification reaction rate, which enhanced the etherification degree and filtration loss performance. When the etherification temperature was 75 °C, the API filtration loss reached the minimum value of 6.32 mL in saturated NaCl brine base slurry at the sample dosage of 1.00% (Figure 8). However, when the etherification temperature was higher than 75 °C, the excessive temperature would increase the rate constants of the side reaction, resulting in a decline to the filtration loss performance of sample [60]. Considering energy consumption and product performance, 75 °C was selected as the optimum etherification temperature.

### 2.3. Comparison between CMCS and Other Fluid Loss Additives

The optimum mass ratio of m_NaOH_:m_MCA_:m_PPS_ was 1:1:2 and the optimum etherification temperature was 75 °C (Figure 6, Figure 7 and Figure 8) The CMCS samples were prepared under this condition. Before and after hot-rolling aging in 4.00% and saturated NaCl brine base slurry for 16 h at 120 °C, the filtration loss tests of PPS, starch, LV-CMC, CMS, and the obtained CMCS were implemented at the sample dosage of 1.50% [59].

**Figure 9:**
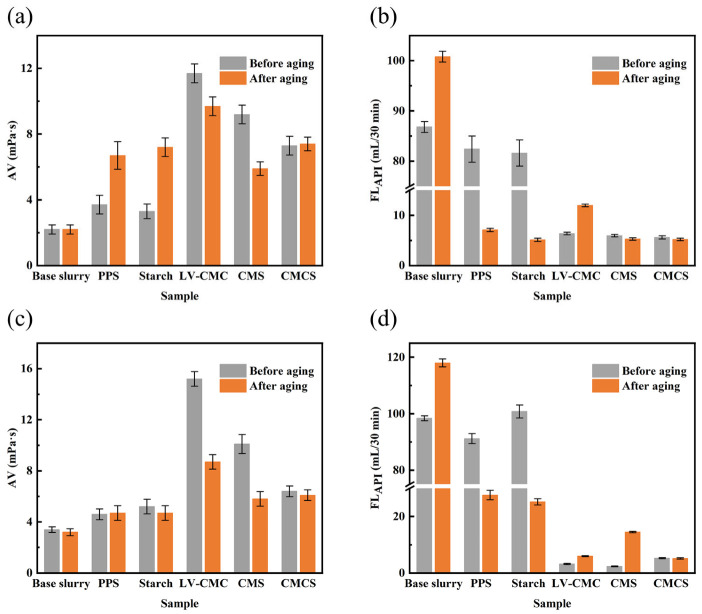
Performances of samples at the sample dosage of 1.50%. (**a**) Apparent viscosity in 4.00% NaCl brine base slurry; (**b**) Filtration loss value in 4.00% NaCl brine base slurry; (**c**) Apparent viscosity in saturated NaCl brine base slurry; (**d**) Filtration loss value in saturated NaCl brine base slurry.

By comparing the filtration loss performance of PPS and starch with its corresponding carboxymethylation product CMCS and CMS, the effect of carboxymethylation reaction can be well proved. Figure 9 indicated that PPS and starch had similar fluid loss reduction performance. With the addition of PPS or starch in 4.00% NaCl and saturated NaCl base slurry, the apparent viscosity and fluid loss value of the drilling fluid hardly changed before aging, which revealed PPS and starch did not have any filtration loss effect before aging. However, after aging at 120 °C for 16 h, obvious changes to the apparent viscosity and fluid loss performance of the drilling fluid could be observed with the addition of PPS or starch, which was due to the gelatinization reaction of starch. When starch was heated in water the surface of starch granules would break, resulting in the exudation of internal soluble substances and the increase in the viscosity. At the same time, some amylopectin oozed out, causing the change in the starch structure, which was beneficial to the fluid loss reduction performance of the drilling fluid [61,62]. After aging, the filtration loss performance of PPS greatly improved in 4.00% NaCl and saturated NaCl brine base slurry. However, the degree of change in saturated NaCl brine base slurry was smaller than that in 4.00% NaCl brine base slurry, which was due to the fact that the tolerance to high NaCl brine concentration of PPS was insufficient. The change trend of the starch after aging was similar to PPS (Figure 9). Therefore, modification should be carried out to PPS and starch to improve their fluid loss performance as a fluid loss agent in water-based drilling fluids.

Figure 9 presented that before and after aging, 4.00% NaCl and saturated NaCl brine base slurry with the addition of LV-CMC or CMS showed good rheological properties and fluid loss performance. LV-CMC and CMS could be used as a good filtrate reducer for drilling fluids; however, they all had their own shortcomings. The fluid loss value of 4.00% NaCl brine base slurry with LV-CMC added was 6.40 mL/30 min and 11.96 mL/30 min before and after aging at 120 °C for 16 h, which indicated that the temperature resistance of LV-CMC was inadequate. After aging at 120 °C for 16 h, the filtration loss value of 4.00% NaCl brine base slurry with the addition of CMS was 5.28 mL/30 min, and it increased to 14.48 mL/30 min in saturated NaCl brine base slurry, which indicated that the resistance to high concentration of NaCl of CMS was insufficient.

With the addition of CMCS, the apparent viscosity of the brine base slurry increased significantly, indicating that the water solubility of CMCS was much higher than that of PPS (Figure 9a,c). Furthermore, the filtration loss performance of CMCS at room temperature was significantly improved compared with that of PPS. When the dosage was 1.50%, the filtration loss value of CMCS and PPS was 5.6 mL/30 min and 82.4 mL/30 min in 4.00% NaCl brine base slurry before aging, respectively. It indicated that the carboxymethylation reaction greatly improved the filtration loss performance of PPS at room temperature. Moreover, CMCS showed stable fluid loss reduction performance in different brine base slurries before and after aging at 120 °C for 16 h, indicating that CMCS had a good resistance to temperature and high concentration of NaCl, which depicted that carboxyl group with resistance to temperature and salt had been introduced successfully in the molecular chain of PPS through carboxymethylation reaction (Figure 9b,d).

When the sample dosage was 1.00%, the filtration loss test was carried out for LV-CMC, CMS, and CMCS in the saturated NaCl brine base slurry before and after aging at 120 °C for 16 h. Table 2 showed the obtained CMCS reached the standard of LV-CMC and modified starch in the specification of drilling fluid material [63].

By comparing the fluid loss reduction performance of LV-CMC, CMS, and CMCS, we could conclude that CMCS had more stable temperature resistance than LV-CMC and had more stable resistance to high concentration of NaCl than CMS. After the pilot scale production of CMCS in the cooperative factory, the price of CMCS was calculated to be about CNY 7000 per ton, which was lower than LV-CMC and CMS. Furthermore, LV-CMC, CMS, and CMCS as drilling fluid additives, were compared in terms of thickening, filtrate loss, salt tolerance, temperature resistance, environmental friendliness, and cost (Table 3).

### 2.4. Rheological Properties of the Drilling Fluid

Drilling fluid is a kind of pseudoplastic fluid, and therefore its rheological properties obey the Power Law equation: ${\tau =K\gamma }^{n}$, where τ is the shear stress, γ is shear rate, *n* is fluidity index, and K is consistency coefficient. K and *n* are two important parameters of pseudoplastic fluid, the value of *n* reflect the shear dilution performance of drilling fluid, and k is related to the viscosity and shear force of drilling fluid. In order to ensure carrying cuttings effectively, the *n* value of drilling fluid is generally required to be around 0.4–0.7. The K value reflects the pumpability of drilling fluid, the large value of K will make it difficult to re-pump, if the K value is too small, it will not be conducive to carrying cuttings. Therefore, the value of K of drilling fluid should also be kept within a proper range. Furthermore, the ratio of yield point to plastic viscosity, which also can reflect the strength of shear dilution, was used to evaluate the rheology behavior of drilling fluid, and is required to be controlled at 0.36–0.48 in drilling fluid technology. Before and after aging, the smaller the changes of the above parameters, the better the high temperature stability of the drilling fluid generally. As is shown in Table 4, the rheological property results of different NaCl concentration brine base slurries with the addition of PPS or CMCS before and after aging were tested and calculated (the dosage was 1.50%). According to the experimental results, different NaCl concentration drilling fluids added with PPS or CMCS have good rheological properties and thermal stability [64,65,66].

### 2.5. Lubricity Performance of CMCS

Before and after aging, the extreme pressure lubrication coefficient reduction rate (Δf) of drilling fluid with different dosages of PPS or CMCS was tested and calculated. Solid lubricants can greatly reduce the friction resistance by changing the sliding friction between drill string and borehole wall into rolling friction, while polymer treatment agents can improve the lubricity of drilling fluid by improving the quality of mud cake and forming adsorption film on drill string and borehole wall [66].

**Figure 10:**
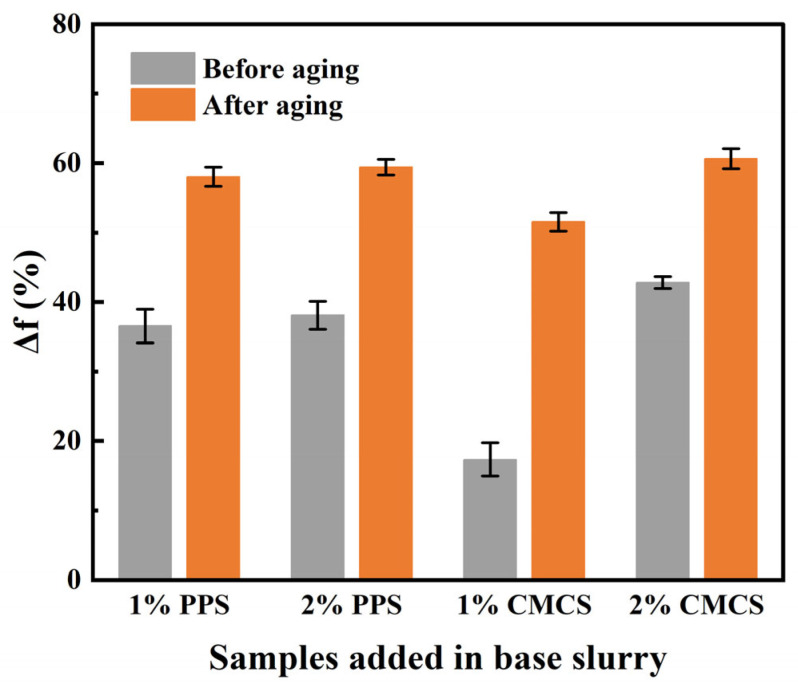
Lubricity performance of PPS and CMCS under different dosages.

Before aging, the lubricity of PPS was better than that of CMCS with 1% addition, because PPS contains many small water-insoluble matters, the sliding friction between the steel ring and the slider will be changed into rolling friction with the addition of PPS, which greatly reduce the friction resistance. However, the strength of adsorption film formed by 1% addition of CMCS is not enough. When the dosage of samples increased to 2%, the strength of the absorption film formed by CMCS was greatly increased, making its Δf increase to 40%, while excessive PPS addition did not improve the lubricity of drilling fluid (Figure 10).

After aging, PPS and CMCS had a better lubricity performance. Under the function of high temperature, the bentonite in the base slurry will agglomerate, resulting in increased viscosity and particle size and reduced lubricity [65,66]. After aging, the adsorption film formed by a water-soluble polymer produced by starch in PPS and its other insoluble small size particles improved the lubricity of the drilling fluid together. Due to the presence of CMCS, the degree of bentonite agglomeration was reduced, and the formed adsorbent film making the drilling fluid had a good lubricity property (Figure 10).

### 2.6. Biodegradability Performance of CMCS

#### 2.6.1. Determination of BOD_5_ of CMCS

The BOD_5_ value of CMCS was determined according to the mentioned method, which was 413 mg/L.

#### 2.6.2. Determination of CODcr of CMCS

The CODcr value of CMCS was calculated according to the results in Table 5 and the calculation formula mentioned above. The experiment was carried out for three times using the potassium dichromate method. The average CODcr value of CMCS was 1600 mg/L.

As a result, the ratio of BOD_5_ and CODcr of the CMCS product was 25.81%, which showed that CMCS is an easily degradable chemical additive and causes no harm to the environment.

### 2.7. Biotoxicity of CMCS

The biological toxicity result of CMCS showed that the EC_50_ value of CMCS was 8.75 × 10^4^ mg/L, which was much higher than the standard requirement value of 2.5 × 10^4^ mg/L. Therefore, CMCS can be used as a non-toxic and eco-friendly filtration reducer for drilling fluids.

## 3. Conclusions

Plant press slag (PPS) is a byproduct of the deep processing of natural polymer products, which is rich in cellulose and starch. A natural mixture of carboxymethyl cellulose and carboxymethyl starch (CMCS) was prepared by alkalization and etherification through PPS and had the optimal filtration loss performance when the mass ratio of m_NaOH_:m_MCA_:m_PPS_ was 1:1:2, and the etherification temperature was 75 °C. After carboxymethylation, the crystallinity of PPS was reduced, and the carboxyl function group was successfully introduced into the molecular chain of PPS. CMCS had a stable filtration loss performance in both 4.00% NaCl and saturated NaCl brine base slurry before and after aging at 120 °C for 16 h. Before aging, the filtration loss value of the obtained CMCS at the dosage of 1.50% in 4.00% NaCl and saturated NaCl brine base slurry was 5.60 mL/30 min and 5.28 mL/30 min, and after aging, the result was 5.20 mL/30 min and 5.28 mL/30 min. Compared with CMS and LV-CMC, CMCS has a better filtration loss performance under high concentration of NaCl and high temperature of 120 °C. The filtration loss performance of CMCS can reach the standards of modified starch and carboxymethyl cellulose for drilling fluids. We can solve the pollution problem of the agricultural waste materials and make full use of them through this chemical modification method. Furthermore, the product CMCS has a good filtration loss performance and it can be used as an environmentally friendly and low-cost filtrate reducer in oilfields.

## 4. Materials and Methods

### 4.1. Materials

**Figure 11:**
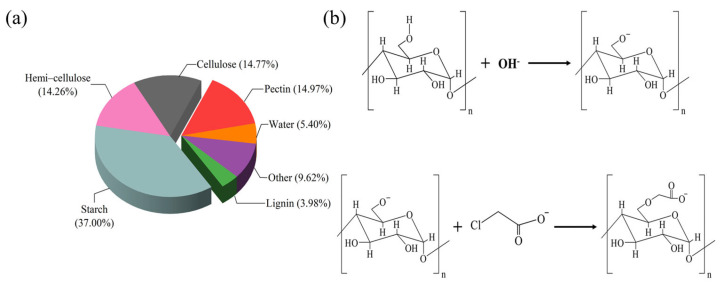
Composition and carboxymethyl reaction mechanism of PPS. (**a**) Compositions of PPS; (**b**) The reaction mechanism of PPS.

Plant press slag (PPS) used in this experiment was a byproduct in the deep processing of natural products and its main components include starch (37.00%), hemi-cellulose (14.26%), cellulose (14.77%), pectin (14.97%), lignin (3.98%), etc., (Figure 11a). The composition analysis of PPS was provided by the Qingdao Institute of Bioenergy and Bioprocess Technology, Chinese Academy of Sciences. PPS was obtained from Inner Mongolia Huaou Starch Industry Co., Ltd. (Hohhot, China).

Technical-grade cotton fiber was obtained from Chenxiang Mining Co., Ltd. (Shijiazhuang, China), and corn starch was obtained from Hexinrong New Materials Co., Ltd. (Renqiu, China). Technical-grade monochloroacetic acid (MCA) was purchased from Yancheng Jinbiao Chemical Industry Co., Ltd. (Yancheng, China). Technical-grade low-viscosity carboxymethyl cellulose (LV-CMC) was obtained from Chongqing Lihong Fine Chemicals Co., Ltd. (Chongqing, China). Sodium carboxymethyl starch (CMS) was obtained from Zhongke Rising Co., Ltd. (Beijing, China). Analytical grade ammonium ferrous sulfate was purchased from Tianjin Guangfu Technology Development Co., Ltd. (Tianjin, China). Other reagents used were analytical grade, which were purchased from Beijing Chemical Factory (Beijing, China) and used without further purification

### 4.2. Methods

#### 4.2.1. Synthesis of CMCS

The PPS was alkalized in the NaOH-ethanol solution of a certain concentration to obtain the alkalized PPS, which included cellulose-Na and starch-Na. CMCS was obtained after etherifying the alkalized PPS by monochloroacetic acid (MCA). Figure 11b showed the reaction mechanism of PPS.

Specific procedures: 20.00 g of PPS and 200.00 mL of 90% ethanol-water solution were added into a three-necked flask with a reflux condenser, a thermometer, and a mechanical stirring device with constant stirring speed. After mixing uniformly, 10.00 g of NaOH (solved in 10 mL distilled water) was added to alkalize PPS for 40 min at room temperature. After alkalization, 10.00 g of MCA (solved in 10 mL distilled water) was added to the container, and the etherification was carried out at 75 °C for 60 min. Finally, the sample was washed 2–3 times with 90% ethanol solution, dried in the oven at 60 °C for 12 h, and smashed to obtain the CMCS.

#### 4.2.2. Elemental Analysis (EA)

The organic elements contents of samples before and after modification were determined by using the organic elemental analyzer (Vario EL cube, Elementar Trading Co., Ltd.; Shanghai, China). The detection limit of the sample was 50 ppm and the accuracy was 0.1 percent.

#### 4.2.3. X-ray Diffraction (XRD)

In order to analyze the crystalline structures, samples were characterized by X-ray Diffraction with Cu Kα radiation (λ = 1.5418 Å), ranging from 10° to 70° (D8 Advance X-ray diffractometer, Bruker Scientific Instruments Hongkong Co. Ltd.; Hongkong, China).

#### 4.2.4. Scanning Electron Microscope (SEM)

A dry sample was mounted on an aluminum holder and an ion sputtering device (Model E-1010, Hitachi; Tokyo, Japan) was used for gold sputter coating to make it be conductive. The morphology of the samples was characterized by a scanning electron microscope (SU8020, Hitachi; Tokyo, Japan) operating at an accelerating voltage of 5 kV.

#### 4.2.5. Fourier Transform Infrared Spectra (FT-IR)

Samples were tested using FT-IR spectrometer (Spectrum 100, PerkinElmer; Waltham, MA, USA) in the range of 4000 cm^−1^ to 500 cm^−1^ with the resolution of 4 cm^−1^ and the signal-noise ratio is 50,000:1. All spectrums were obtained by accumulating 64 scans.

#### 4.2.6. Thermogravimetric (TG)

The thermal decomposition behavior of the samples was investigated using thermogravimetric analysis (STA449F3; Netzsch; Selb, Germany) under the nitrogen (N_2_) atmosphere and the nitrogen flow rate was 40 mL/min. The samples were placed in a clean crucible and heated from 30 °C to 600 °C at a heating rate of 10 °C/min. Differential thermogravimetric (DTG) analysis curves were obtained with the TGA data by numerically differentiating the latter with respect to temperature.

#### 4.2.7. X-ray Photoelectron Spectroscopy (XPS)

The elements’ binding energies of samples before and after modification were carried out using X-ray photoelectron spectroscopy (XPS, Escalab 250Xi, Thermo Fisher Scientific; Waltham, MA, USA). A deconvolution curve fitting was performed for the C_1s_ peaks, and the spectra were fitted using the Gaussian peak profiles and a linear background.

#### 4.2.8. Performance Tests of the Drilling Fluids

**Figure 12:**
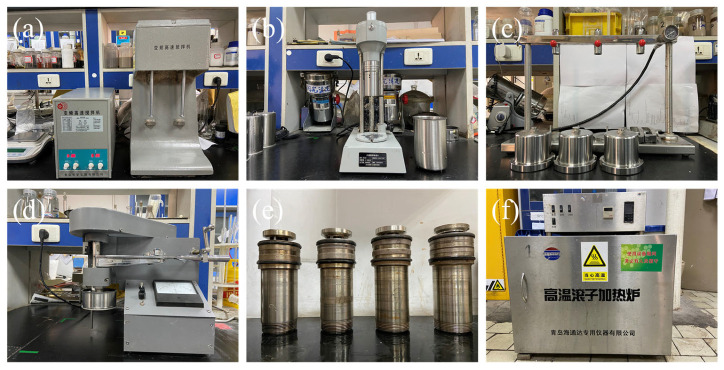
Instruments used during the experiment. (**a**) High-speed mixer; (**b**) Six-speed rotational viscometer; (**c**) Medium-pressure filter press; (**d**) Extreme pressure and lubricity tester; (**e**) High-temperature aging tank; (**f**) High-temperature hot-rolling furnace.

Tests of drilling fluid performances included API filtration loss, temperature tolerance, lubricity, and rheological properties. Evaluation methods on drilling fluid performances were carried out in accordance with API and Sinopec group recommended standard method [59]. The experimental apparatus used are shown in Figure 12.

##### Preparation of Base Slurry

(1) In total, 4.00% NaCl brine base slurry: the base slurry was prepared following the ratio of 100.00 g of England Evaluation Clay (a famous standard kaolin clay from Britain), 40.00 g of NaCl and 2.80 g of NaHCO_3_ in 1 L of distilled water. The mixture was stirred for 20 min at high speed, and at least two stops were needed during the stirring to scrape the clay adhered on the container wall. Finally, sealed and conserved at 25 ± 3 °C for 24 h. (2) Saturated NaCl brine base slurry: the base slurry was prepared following the ratio of 100.00 g of England Evaluation Clay, 365.00 g of NaCl and 2.80 g of NaHCO_3_ in 1 L of distilled water. The mixture was stirred for 20 min at high speed, and at least two stops were needed during the stirring to scrape the clay adhered on the container wall. Finally, sealed and conserved at 25 ± 3 °C for 24 h.

##### Determination of API Filtration Loss

The API filtration loss was determined on a medium-pressure filter press (SD3, Qingdao Tongchun Oil Instrument Co. Ltd.; Qingdao, China). A certain amount of sample was added into 400 mL of brine base slurry and stirred at high speed. After 20 min, the mixture was poured into a sealed container and cured at room temperature. After 16 h, the drilling fluid was stirred at a high speed for 5 min, and poured into the filtration instrument cup up to the scale mark. The filtrate was collected under 0.69 MPa in 30 min. The volume of the obtained filtrate represents the API filtration loss (FL_API_) of the drilling fluid.

##### Determination of Temperature Tolerance

The mixture of a certain amount of sample and 400 mL of brine base slurry were stirred at high speed for 20 min, poured into a high-temperature aging tank and placed it in high-temperature hot-rolling furnace (Hot roll furnace, XGRL-5, Qingdao Haitongda Special Instrument Co., Ltd.; Qingdao, China) to age under a certain temperature for 16 h. After 16 h, the drilling fluid was cooled down to room temperature and stirred for 5 min at high speed, and then the filtration loss and rheological property were tested.

##### Determination of Rheological Property

A certain amount of sample was added into 400 mL of the brine base slurry, which was closely conserved for at least 24 h. The mixture was stirred for 20 min at high speed and poured into the sample cup of Model Fanns 35 6-speed rotational viscometer (ZNN-D6, Qingdao Haitongda Special Instrument Co., Ltd.; Qingdao, China). The liquid level of the sample cup is tangent to the scale mark of drum of the 6-speed rotational viscometer and the stabilized values at different speeds were recorded. The apparent viscosity (AV), plastic viscosity (PV), yield point (YP), ration of yield point to plastic viscosity (RYP), the fluidity index (*n*), and consistency coefficient (K) of power law model fluid equation were calculated according to the following formulas:

(1) $$\mathrm{AV}=0.5\times \Phi_{600}$$

(2) $$\mathrm{PV}=\Phi_{600}-\Phi_{300}$$

(3) $$\mathrm{YP}=0.511\left(\Phi_{300}-\mathrm{PV}\right)$$

(4) RYP=YP/PV

(5) $$n=3.322lg\left(\Phi_{600}/\Phi_{300}\right)$$

(6) $$K=\left(0.511\Phi_{300}\right)/511^{n}$$

where:

AV is the apparent viscosity (mPa·s);

PV is the plastic viscosity (mPa·s);

YP is the yield point (Pa);

RYP is the ratio of yield point to plastic viscosity (mPa·s);

Φ_600_ is the dial reading of 6-speed rotational viscometer at 600 r/min (dia);

Φ_300_ is the dial reading of 6-speed rotational viscometer at 300 r/min (dia); 

*n* is the fluidity index of power law model rheological equation (dimensionless quantity);

K is the consistency coefficient of power law model rheological equation (Pa·s^*n*^).

##### Determination of Lubricity Performance

The evaluation methods are according to the Sinopec enterprise standard, Q/SH1020 1879–2016 [67]. The extreme pressure lubrication coefficient reduction rate (Δf) was used to evaluate the lubrication performance with extreme pressure (E-P) and lubricity tester (Fann Model 212, Fann Instrument Company; Houston, TX, USA). The test steps were as follows: (1) The extreme pressure lubricator was turned on and preheated for 15 min. (2) The test slider was secured on the bracket and attached to the test ring. (3) The rotation speed of the test ring was adjusted to 60 rpm and torque was adjusted to zero. (4) The cured 400 mL brine base slurry was stirred at high speed for 5 min, and then poured into the test tray to the scale mark. Then, the torque was adjusted to 16.95 N∙m, and the reading was recorded as *T*_1_ after testing for 10 min. (5) The cured mixture of a certain amount of sample and 400 mL brine base slurry was stirred at high speed for 5 min. After cleaning the test slider and ring, the mixture was carried out using the same operation in step 4, and the reading was recorded as *T*_2_ after testing for 10 min. Then, Δf was calculated according to the following formula:

(7) $$\Delta f=\frac{\left(T_{1}-T_{2}\right)}{T_{1}}\times 100$$

where:

Δf is the extreme pressure lubrication coefficient reduction rate of the samples (%); 

*T*_1_ is the extreme pressure lubrication coefficient of the base slurry; 

*T*_2_ is the extreme pressure lubrication coefficient of the slurry added with samples.

#### 4.2.9. Biodegradability Test

The biodegradable performance of the sample is evaluated by the ratio of Biological Oxygen Demand (BOD_5_) and Chemical Oxygen Demand (CODcr).

##### Determination of BOD_5_

The BOD_5_ value was determined by measuring the consumed oxygen in the sample decomposition process under certain experimental conditions. The sample was pretreated according to Chinese enterprise standard, HJ 557-2010 [68]. Then, the sample solution was analyzed with a BOD5 tester (JC-870H, Qingdao Juchuang Times Environmental Protection Technology Co., Ltd.; Qingdao, China) and the instrument reading was recorded.

##### DDetermination of CODcr

The CODcr of sample was determined with potassium dichromate method according to the Chinese national standard, GB/11914-89 [69]. A certain amount of sample was added into a conical flask and oxidized by appropriate amount of potassium bichromate standard solution under the catalysis of sulfuric acid-silver sulfate solution. After heated and reflux for a period of time, the ammonium ferrous sulfate standard solution was used for titration, with 1,10-Phenanthroline as an indicator, and the volume of consumed ammonium ferrous sulfate standard solution was recorded as V_2_. Take the same amount of distilled water from the above experimental conditions, and the amount of consumed ammonium ferrous sulfate standard solution of the blank group was recorded as V_1_. Then, the CODcr (mg/L) was calculated according to the following formula:

(8) $$\mathrm{COD}(\mathrm{mg}/L)=\frac{c\left(V_{1}-V_{2}\right)\times 8000}{V_{0}}$$

where:

*c* is the concentration of ammonium ferrous sulfate standard solution (mol/L); 

V_1_ is the volume of consumed ammonium ferrous sulfate standard solution of the blank group (mL); 

V_2_ is the volume of consumed ammonium ferrous sulfate standard solution of the sample (mL); 

V_0_ is the volume of sample solution (mL).

#### 4.2.10. Biological Toxicity Test

The biological toxicity tests were carried out according to the CNPC enterprise standard, Q/SY 111-2007 [70]. The clear sample solution was added into the biotoxicity tester (Microtox LX, Modern Water, Shanghai, China), and when the strength of luminescent bacterial luminous became 50%, the concentration of the solution was determined. The value of EC_50_ was calculated according to the standard method by the instrument display value.

## Figures and Tables

**Table 1:** Elemental analysis results of PPS and CMCS.

Sample	Weight/mg	C/%	H/%	O/%	C/H	C/O
PPS	2.075	38.560	5.667	48.062	6.804	0.802
CMCS	2.139	32.170	4.691	54.241	6.858	0.593

**Table 2:** Contrast of LV-CMC, CMS, and CMCS according to the standards in the specification of drilling fluid materials.

Filtrate Reducer	FL_API_ in Saturated Brine Base Slurry (mL/30 min)		Market Price (CNY/t)
	Before Aging	After Aging	
LV-CMC	6.0	16.0	11,500
CMS	4.0	34.0	9000
CMCS	6.4	7.0	7000
Standards	≤10.00	-	-

**Table 3:** Comparison results of LV-CMC, CMS, and CMCS.

Index	LV-CMC	CMS	CMCS
Thickening	ΔΔΔ	ΔΔ	ΔΔ
Filtrate loss	ΔΔΔ	ΔΔΔ	ΔΔΔ
Salt tolerance	ΔΔΔ	Δ	ΔΔΔ
Temperature resistance	ΔΔ	Δ	ΔΔ
Environmental friendliness	ΔΔΔ	ΔΔΔ	ΔΔΔ
Value for money	Δ	ΔΔ	ΔΔΔ

The number of Δ indicates the degree of excellence of an index.

**Table 4:** Rheological property results of the drilling fluids with the addition of PPS or CMCS.

T/℃	Index	4% NaCl Slurry		Saturated NaCl Slurry	
		with PPS	with CMCS	with PPS	with CMCS
25	AV/mPa·s	4	8	5	7
	PV/mPa·s	3	6	3	5
	RYP/(Pa/mPa·s)	0.3407	0.3407	0.6813	0.4088
	*n*/Dimensionless	0.6781	0.6781	0.5146	0.6374
	K/Pa·s^*n*^	0.0372	0.0744	0.1445	0.0864
120	AV/mPa·s	7	7.5	4.5	6
	PV/mPa·s	6	5	3	5
	RYP/(Pa/mPa·s)	0.1703	0.5110	0.5110	0.2044
	*n*/Dimensionless	0.8074	0.5850	0.5850	0.7776
	K/Pa·s^*n*^	0.0266	0.1330	0.0789	0.0280

**Table 5:** The volume of consumed ammonium ferrous sulfate standard solution.

	Dilution Rate	Test 1	Test 2	Test 3	Average Value
V_1_/mL	50	19.50	19.20	19.10	19.27
V_2_/mL	50	18.50	18.40	18.50	18.47

## Data Availability

Data are available from the authors upon request.
